# Bis(3-methyl­anilinium) hexa­chlorido­stannate(IV) dihydrate

**DOI:** 10.1107/S1600536812017618

**Published:** 2012-04-28

**Authors:** Ming-Liang Liu

**Affiliations:** a Ordered Matter Science Research Center, Southeast University, Nanjing 211189, People’s Republic of China

## Abstract

In the title compound, (C_7_H_10_N)_2_[SnCl_6_]·2H_2_O, the Sn^IV^ atom lies on a site with symmetry 2/*m*. One of the Cl atoms lies on a mirror plane and the 3-methyl­anilinium cation is also situated on a mirror plane. The water mol­ecule is located on a twofold rotation axis. The H atoms of the methyl and ammonium groups and the solvent water mol­ecule are disordered by symmetry. In the crystal, N—H⋯Cl, O—H⋯Cl and N—H⋯O hydrogen bonds connect the organic cations, the inorganic octahedrally shaped anions and the water mol­ecules.

## Related literature

For background to ferroelectric metal-organic complexes, see: Zhang *et al.* (2009 ▶, 2010 ▶). For related structures, see: Liu (2011*a* ▶,*b* ▶,*c* ▶).
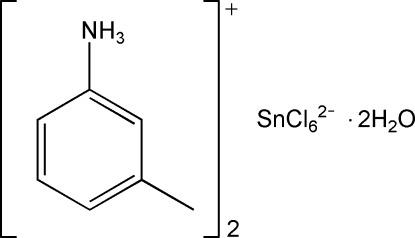


## Experimental

### 

#### Crystal data

(C_7_H_10_N)_2_[SnCl_6_]·2H_2_O
*M* *_r_* = 583.74
Monoclinic, 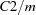

*a* = 20.467 (4) Å
*b* = 7.1699 (14) Å
*c* = 7.7569 (16) Å
β = 93.83 (3)°
*V* = 1135.8 (4) Å^3^
*Z* = 2
Mo *K*α radiation
μ = 1.84 mm^−1^
*T* = 293 K
0.36 × 0.32 × 0.28 mm

#### Data collection

Rigaku Mercury2 CCD diffractometer
Absorption correction: multi-scan (*CrystalClear*; Rigaku, 2005 ▶) *T*_min_ = 0.963, *T*_moax_ = 0.971
5833 measured reflections
1405 independent reflections
1370 reflections with *I* > 2σ(*I*)
*R*_int_ = 0.036

#### Refinement

*R*[*F* ^2^ > 2σ(*F* ^2^)] = 0.030
*wR*(*F* ^2^) = 0.096
*S* = 0.92
1405 reflections
74 parameters
H-atom parameters constrained
Δρ_max_ = 0.38 e Å^−3^
Δρ_min_ = −0.73 e Å^−3^

### 

Data collection: *CrystalClear* (Rigaku, 2005 ▶); cell refinement: *CrystalClear*; data reduction: *CrystalClear*; program(s) used to solve structure: *SHELXS97* (Sheldrick, 2008 ▶); program(s) used to refine structure: *SHELXL97* (Sheldrick, 2008 ▶); molecular graphics: *SHELXTL* (Sheldrick, 2008 ▶); software used to prepare material for publication: *SHELXTL*.

## Supplementary Material

Crystal structure: contains datablock(s) I, global. DOI: 10.1107/S1600536812017618/hy2538sup1.cif

Structure factors: contains datablock(s) I. DOI: 10.1107/S1600536812017618/hy2538Isup2.hkl

Additional supplementary materials:  crystallographic information; 3D view; checkCIF report

## Figures and Tables

**Table 1:** Hydrogen-bond geometry (Å, °)

*D*—H⋯*A*	*D*—H	H⋯*A*	*D*⋯*A*	*D*—H⋯*A*
N1—H1*A*⋯Cl2^i^	0.89	2.59	3.476 (4)	171
N1—H1*B*⋯O1^ii^	0.89	1.93	2.809 (5)	170
N1—H1*C*⋯Cl1^iii^	0.89	2.75	3.5883 (7)	157
O1—H1*D*⋯Cl2	0.85	2.44	3.228 (2)	154

Symmetry codes: (i) 
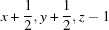
; (ii) 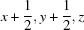; (iii) 
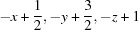
.

**Fig. 1.**
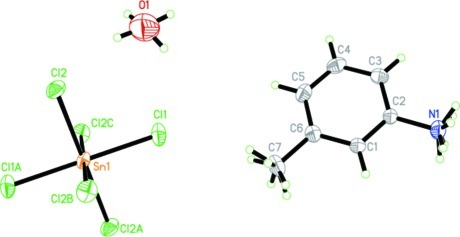
The molecular structure of the title compound. Displacement ellipsoids are drawn at the 30% probability level. H atoms on C7, N1 and O1 are disordered over two sets of sites. [Symmetry codes: (A) -x, -y, 2-z; (B) -x, y, 2-z; (C) x, -y, z.]

## supplementary crystallographic information

### Comment

Recently much attention has been devoted to metal-organic compounds due to the tunability of their special structural features and their interesting physical properties (Zhang *et al.*, 2009, 2010). As a continuation of our researches (Liu, 2011*a*,b,c), the title compound has been synthesized and its crystal structure is herein reported.

In the title compound, the Sn^IV^ atom lies on a 2/m symmetry site, and is coordinated by six Cl atoms (Fig. 1). One of the Cl atoms lies on a mirror plane and the 3-methylanilinium cation is also situated on a mirror plane. The water molecule is located on a twofold rotation axis. The H atoms of the methyl and amidogen groups and the water molecule are disordered induced by symmetry. N—H···Cl, O—H···Cl and N—H···O hydrogen-bonding interactions connect the [SnCl_6_]^2-^ anions, the 3-methylanilinium cations and the water molecules (Table 1). The non-H atoms of the 3-methylanilinium cation are coplanar. The average Sn—Cl bond distances range from 2.4260 (13) to 2.4384 (9) Å and the *cis* Cl—Sn—Cl angles range from 88.78 (5) to 91.22 (5)°.

### Experimental

3-Methylbenzenamine (3.21 g, 0.03 mol) was dissolved in 30 ml ethanol, to which hydrochloric acid (1.1 g, 0.03 mol) was then added. Stannous chloride (2.25 g, 0.01 mol) was dissolved in 20 ml ethanol, to which was added hydrochloric acid, then mixed with the above solution without any precipitation under stirring at ambient temperature. Single crystals suitable for X-ray structure analysis were obtained by slow evaporation after 4 days in air.

The dielectric constant of the compound as a function of temperature indicates that the permittivity is basically temperature-independent [*ε* = C/(T–T_0_)], suggesting that this compound is not ferroelectric or there may be no distinct phase transition occurring within the measured temperature (below the melting point).

### Refinement

H atoms on C atoms were positioned geometrically and refined as riding atoms, with C—H = 0.93 (aromatic), 0.96 (methyl) and N—H = 0.89 Å and with *U*_iso_(H) = 1.2*U*_eq_(C, N). Water H atoms were located from a difference Fourier map and refined as riding atoms, with O—H = 0.85 Å and *U*_iso_(H) = 1.5*U*_eq_(O).

### Crystal data

**Table:**

(C_7_H_10_N)_2_[SnCl_6_]·2H_2_O	*F*(000) = 580
*M**_r_* = 583.74	*D*_x_ = 1.707 Mg m^−^^3^
Monoclinic, *C*2/*m*	Mo *K*α radiation, λ = 0.71073 Å
Hall symbol: -C 2y	Cell parameters from 1370 reflections
*a* = 20.467 (4) Å	θ = 3.4–25.0°
*b* = 7.1699 (14) Å	µ = 1.84 mm^−^^1^
*c* = 7.7569 (16) Å	*T* = 293 K
β = 93.83 (3)°	Block, colourless
*V* = 1135.8 (4) Å^3^	0.36 × 0.32 × 0.28 mm
*Z* = 2	

### Data collection

**Table:**

Rigaku Mercury2 CCD diffractometer	1405 independent reflections
Radiation source: fine-focus sealed tube	1370 reflections with *I* > 2σ(*I*)
Graphite monochromator	*R*_int_ = 0.036
ω scans	θ_max_ = 27.5°, θ_min_ = 3.0°
Absorption correction: multi-scan (*CrystalClear*; Rigaku, 2005)	*h* = −26→25
*T*_min_ = 0.963, *T*_max_ = 0.971	*k* = −9→9
5833 measured reflections	*l* = −10→10

### Refinement

**Table:**

Refinement on *F*^2^	Secondary atom site location: difference Fourier map
Least-squares matrix: full	Hydrogen site location: inferred from neighbouring sites
*R*[*F*^2^ > 2σ(*F*^2^)] = 0.030	H-atom parameters constrained
*wR*(*F*^2^) = 0.096	*w* = 1/[σ^2^(*F*_o_^2^) + (0.0749*P*)^2^ + 1.7826*P*] where *P* = (*F*_o_^2^ + 2*F*_c_^2^)/3
*S* = 0.92	(Δ/σ)_max_ < 0.001
1405 reflections	Δρ_max_ = 0.38 e Å^−^^3^
74 parameters	Δρ_min_ = −0.73 e Å^−^^3^
0 restraints	Extinction correction: *SHELXL97* (Sheldrick, 2008), Fc^*^=kFc[1+0.001xFc^2^λ^3^/sin(2θ)]^-1/4^
Primary atom site location: structure-invariant direct methods	Extinction coefficient: 0.041 (2)

### Special details

**Table:**

Geometry. All e.s.d.'s (except the e.s.d. in the dihedral angle between two l.s. planes) are estimated using the full covariance matrix. The cell e.s.d.'s are taken into account individually in the estimation of e.s.d.'s in distances, angles and torsion angles; correlations between e.s.d.'s in cell parameters are only used when they are defined by crystal symmetry. An approximate (isotropic) treatment of cell e.s.d.'s is used for estimating e.s.d.'s involving l.s. planes.
Refinement. Refinement of *F*^2^ against ALL reflections. The weighted *R*-factor *wR* and goodness of fit *S* are based on *F*^2^, conventional *R*-factors *R* are based on *F*, with *F* set to zero for negative *F*^2^. The threshold expression of *F*^2^ > σ(*F*^2^) is used only for calculating *R*-factors(gt) *etc*. and is not relevant to the choice of reflections for refinement. *R*-factors based on *F*^2^ are statistically about twice as large as those based on *F*, and *R*- factors based on ALL data will be even larger.

### Fractional atomic coordinates and isotropic or equivalent isotropic displacement parameters (Å^2^)

**Table:**

	*x*	*y*	*z*	*U*_iso_*/*U*_eq_	Occ. (<1)
N1	0.4203 (2)	1.0000	0.2314 (5)	0.0565 (10)	
H1A	0.4253	0.9464	0.1296	0.068*	0.50
H1B	0.4429	0.9367	0.3140	0.068*	0.50
H1C	0.4349	1.1169	0.2294	0.068*	0.50
C1	0.3335 (2)	1.0000	0.4366 (5)	0.0416 (8)	
H1	0.3661	1.0000	0.5261	0.050*	
C2	0.3501 (2)	1.0000	0.2664 (5)	0.0392 (8)	
C3	0.3029 (2)	1.0000	0.1309 (5)	0.0537 (11)	
H3	0.3147	1.0000	0.0171	0.064*	
C4	0.2382 (2)	1.0000	0.1673 (6)	0.0618 (13)	
H4	0.2058	1.0000	0.0774	0.074*	
C5	0.2208 (2)	1.0000	0.3365 (7)	0.0542 (11)	
H5	0.1767	1.0000	0.3588	0.065*	
C6	0.2677 (2)	1.0000	0.4732 (5)	0.0429 (8)	
C7	0.2496 (3)	1.0000	0.6588 (7)	0.0622 (13)	
H7A	0.2819	1.0687	0.7285	0.075*	0.50
H7B	0.2481	0.8739	0.7000	0.075*	0.50
H7C	0.2075	1.0574	0.6659	0.075*	0.50
Sn1	0.0000	0.0000	1.0000	0.0370 (2)	
Cl1	0.07220 (7)	0.0000	0.76437 (18)	0.0617 (3)	
Cl2	−0.06652 (4)	0.24302 (12)	0.85527 (11)	0.0565 (3)	
O1	0.0000	0.3385 (11)	0.5000	0.136 (3)	
H1D	−0.0216	0.3511	0.5891	0.203*	0.50
H1E	0.0390	0.3062	0.5304	0.203*	0.50

### Atomic displacement parameters (Å^2^)

**Table:**

	*U*^11^	*U*^22^	*U*^33^	*U*^12^	*U*^13^	*U*^23^
N1	0.0412 (19)	0.081 (3)	0.0478 (19)	0.000	0.0093 (15)	0.000
C1	0.0394 (19)	0.050 (2)	0.0354 (17)	0.000	−0.0004 (14)	0.000
C2	0.0346 (18)	0.046 (2)	0.0368 (17)	0.000	0.0041 (14)	0.000
C3	0.054 (3)	0.072 (3)	0.0344 (18)	0.000	−0.0035 (17)	0.000
C4	0.045 (2)	0.085 (4)	0.053 (2)	0.000	−0.0157 (19)	0.000
C5	0.035 (2)	0.063 (3)	0.064 (3)	0.000	0.0028 (18)	0.000
C6	0.047 (2)	0.0370 (19)	0.0458 (19)	0.000	0.0089 (16)	0.000
C7	0.070 (3)	0.065 (3)	0.054 (2)	0.000	0.025 (2)	0.000
Sn1	0.0290 (2)	0.0291 (2)	0.0536 (3)	0.000	0.00791 (15)	0.000
Cl1	0.0550 (7)	0.0597 (7)	0.0742 (7)	0.000	0.0323 (6)	0.000
Cl2	0.0503 (4)	0.0477 (4)	0.0712 (5)	0.0134 (3)	0.0023 (3)	0.0088 (4)
O1	0.127 (5)	0.173 (7)	0.105 (4)	0.000	−0.009 (4)	0.000

### Geometric parameters (Å, º)

**Table:**

N1—C2	1.480 (5)	C5—H5	0.9300
N1—H1A	0.8900	C6—C7	1.511 (6)
N1—H1B	0.8899	C7—H7A	0.9602
N1—H1C	0.8901	C7—H7B	0.9600
C1—C2	1.385 (5)	C7—H7C	0.9600
C1—C6	1.395 (6)	Sn1—Cl1^i^	2.4260 (13)
C1—H1	0.9300	Sn1—Cl1	2.4260 (13)
C2—C3	1.380 (6)	Sn1—Cl2^ii^	2.4384 (9)
C3—C4	1.372 (7)	Sn1—Cl2^iii^	2.4384 (9)
C3—H3	0.9300	Sn1—Cl2	2.4384 (9)
C4—C5	1.383 (7)	Sn1—Cl2^i^	2.4384 (9)
C4—H4	0.9300	O1—H1D	0.8500
C5—C6	1.383 (7)	O1—H1E	0.8499
			
C2—N1—H1A	109.5	C1—C6—C7	119.7 (4)
C2—N1—H1B	109.4	C6—C7—H7A	109.5
H1A—N1—H1B	109.5	C6—C7—H7B	109.5
C2—N1—H1C	109.5	H7A—C7—H7B	109.4
H1A—N1—H1C	109.5	C6—C7—H7C	109.4
H1B—N1—H1C	109.5	H7A—C7—H7C	109.5
C2—C1—C6	119.7 (4)	H7B—C7—H7C	109.5
C2—C1—H1	120.2	Cl1^i^—Sn1—Cl1	180.0
C6—C1—H1	120.2	Cl1^i^—Sn1—Cl2^ii^	89.85 (4)
C3—C2—C1	121.5 (4)	Cl1—Sn1—Cl2^ii^	90.15 (3)
C3—C2—N1	119.9 (4)	Cl1^i^—Sn1—Cl2^iii^	90.15 (4)
C1—C2—N1	118.5 (4)	Cl1—Sn1—Cl2^iii^	89.85 (4)
C2—C3—C4	118.6 (4)	Cl2^ii^—Sn1—Cl2^iii^	180.0
C2—C3—H3	120.7	Cl1^i^—Sn1—Cl2	89.85 (3)
C4—C3—H3	120.7	Cl1—Sn1—Cl2	90.15 (3)
C5—C4—C3	120.7 (4)	Cl2^ii^—Sn1—Cl2	91.22 (5)
C5—C4—H4	119.7	Cl2^iii^—Sn1—Cl2	88.78 (5)
C3—C4—H4	119.7	Cl1^i^—Sn1—Cl2^i^	90.15 (3)
C6—C5—C4	121.1 (4)	Cl1—Sn1—Cl2^i^	89.85 (3)
C6—C5—H5	119.5	Cl2^ii^—Sn1—Cl2^i^	88.78 (5)
C4—C5—H5	119.5	Cl2^iii^—Sn1—Cl2^i^	91.22 (5)
C5—C6—C1	118.4 (4)	Cl2—Sn1—Cl2^i^	180.0
C5—C6—C7	121.9 (4)	H1D—O1—H1E	109.5
			
C6—C1—C2—C3	0.0	C3—C4—C5—C6	0.0
C6—C1—C2—N1	180.0	C4—C5—C6—C1	0.0
C1—C2—C3—C4	0.0	C4—C5—C6—C7	180.0
N1—C2—C3—C4	180.0	C2—C1—C6—C5	0.0
C2—C3—C4—C5	0.0	C2—C1—C6—C7	180.0

Symmetry codes: (i) −*x*, −*y*, −*z*+2; (ii) *x*, −*y*, *z*; (iii) −*x*, *y*, −*z*+2.

### Hydrogen-bond geometry (Å, º)

**Table:**

*D*—H···*A*	*D*—H	H···*A*	*D*···*A*	*D*—H···*A*
N1—H1*A*···Cl2^iv^	0.89	2.59	3.476 (4)	171
N1—H1*B*···O1^v^	0.89	1.93	2.809 (5)	170
N1—H1*C*···Cl1^vi^	0.89	2.75	3.5883 (7)	157
O1—H1*D*···Cl2	0.85	2.44	3.228 (2)	154

Symmetry codes: (iv) *x*+1/2, *y*+1/2, *z*−1; (v) *x*+1/2, *y*+1/2, *z*; (vi) −*x*+1/2, −*y*+3/2, −*z*+1.
